# Supplementary material for: The Influence of Ultra-Low Tidal Volume Ventilation during Cardiopulmonary Resuscitation on Renal and Hepatic End-Organ Damage in a Porcine Model
Source: Biomedicines. 2023 Mar 14;11(3):899. doi: 10.3390/biomedicines11030899 (PMC10045409; doi:10.3390/biomedicines11030899)
Supplement: Supplementary file 1 [file biomedicines-11-00899-s001.zip › biomedicines-2235349 - supplementary .pdf]

biomedicines

MDPI

Article

The Influence of Ultra-Low Tidal Volume Ventilation During Cardiopulmonary Resuscitation on Renal and Hepatic End-Organ Damage in a Porcine Model

Katja Mohnke [1*], Victoria Buschmann [1], Thomas Baller [1], Julian Riedel [1], Miriam Renz [1], René Rissel [1], Alexander Ziebart [1], Erik K. Hartmann [1] and Robert Ruemmler [1]

Medical Center, Department of Anaesthesiology, Johannes Gutenberg University, Langenbeckstr. 1, 55131 Mainz, Germany
* Correspondence: katja.mohnke@uni-mainz.de

Table S1. Grading of histopathological liver damage. The assessment criteria are shown, which were used to classify the six-level damage severity of the 5 items. The term "zone" refers to the metabolic zones of the liver acini [15].

Subitem	Grading of Severity					
	0	1	2	3	4	5
**Centrilobular Necrosis**	No pathological changes	Incipient necrotic changes in individual hepatocytes	Isolated necrosis in the area of the central veins	Centrilobular necrosis limited to zone 3 in some liver lobules	Centrilobular necrosis up to zone 2 in many liver lobules	Extensive centrilobular necrosis over all zones in most lobules of the liver
**Hydropic Cell Swelling**	No pathological changes	Isolated incipient cell swelling	Cell swelling of several individual hepatocytes	Cell swelling of neighboring hepatocytes with rarefaction of the cell plasma	Pronounced hydropic cell swelling in several areas of the liver lobules	Pronounced hydropic cell swelling over extensive areas of the liver lobules with marked decrease in stainability
**Inflammation**	Scattered immune cells in the blood vessels	Increased immune cells in the blood vessels with transfer into the tissue, activated local Kupffer cells	Increased occurrence of immune cells in the area of the central veins	Increased occurrence of immune cells up to zone 3	Increased occurrence of immune cells up to zone 2	Generalized inflammation of the liver lobules

**Venous Congestion**	No signs of venous congestion	Isolated dilated central veins without compression of the adjacent tissue	Multiple dilated central veins without compression of adjacent tissue	Hyperemic with a plurality of dilated central veins	Hyperemic with multiple dilated central veins and compression of adjacent tissue	Pronounced hyperemic with dilatation of almost all central veins
**Sinusoidal Congestion**	No evidence of sinusoidal congestion	Scattered dilatation of sinuoids adjacent to central veins	Scattered enlargement of sinuoids over zone 3	Increased enlargement of sinuoids over zone 2	Pronounced enlargement of sinuoids with hepatocytes drifting apart and accumulation of erythrocytes in zone 1	Pronounced enlargement of sinuoids with hepatocytes drifting apart and congestion of erythrocytes in all zones

Disclaimer/Publisher's Note: The statements, opinions and data contained in all publications are solely those of the individual author(s) and contributor(s) and not of MDPI and/or the editor(s). MDPI and/or the editor(s) disclaim responsibility for any injury to people or property resulting from any ideas, methods, instructions or products referred to in the content.
